# Mycobiota dysbiosis and gastric tumorigenesis

**DOI:** 10.7150/thno.61480

**Published:** 2021-06-01

**Authors:** Nicolas Papon, Tobias M. Hohl, Bing Zhai

**Affiliations:** 1 Univ Angers, Univ Brest, GEIHP, SFR ICAT, F-49000 Angers, France.; 2 Infectious Disease Service, Department of Medicine, Memorial Sloan Kettering Cancer Center, New York, NY, USA.; 3 Immunology Program, Sloan Kettering Institute, Memorial Sloan Kettering Cancer Center, New York, NY, USA.; 4 Weill Cornell Medical College, New York, NY, USA.

**Keywords:** Fungi, mycobiota, Candida, cancer, dysbiosis

## Abstract

The gastrointestinal tract contains a vast and diverse microbial reservoir composed of bacteria, fungi, and viruses that contribute positively to human health. There is growing evidence that perturbation of the normal microbiota can promote a variety of human disease states that include tumorigenesis. Whether the fungal component of the gut microbiota (i.e., the mycobiota) can influence tumor development has not been investigated in detail. In the recent issue of the *Theranostics*, Zhong et al (2021) shed light on an association between mycobiota dysbiosis and gastric cancer. These findings implicate the mycobiota in gastric carcinogenesis and set the stage for future mechanistic studies to explore whether fungal dysbiosis is a cause or consequence of gastric carcinogenesis, with important implications for preventative strategies.

Cancer remains the second leading cause of death worldwide after heart disease. While lung cancer claims the most lives worldwide, gastric cancer (GC) ranks fourth with over one million new cases and 600,000-700,000 deaths globally each year 1. There is growing evidence that the microbiota plays an important role in influencing gastrointestinal carcinogenesis 2, 3, in particular bacterial components that could either protect against or enhance tumorigenesis. For instance, *Helicobacter pylori*, long associated with the development of peptic ulcer disease, acts as a carcinogen that increases gastric cancer risk. The importance of the mycobiota in gastrointestinal cancer development is poorly understood, though recent studies implicate gut fungi in pancreatic, oesophageal, and colonic oncogenesis 4-7. In this issue of *Theranostics*, Zhong and colleagues report an association between mycobiota dysbiosis and GC 8 (Figure 1).

In this study, the research groups of Yongxi Song and Xuehui Hong took advantage of surgical biopsies that included cancerous lesions and adjacent non-cancerous tissues and were obtained from 45 patients admitted at the First Affiliated Hospital of China Medical University, Shenyang, China. The authors used non-culture based high-throughput amplicon sequencing to characterize fungal populations in diseased and healthy tissues. First, their results indicated that Ascomycota, and to a lesser extent Basidiomycota, were the most abundant fungal phyla represented in both GC and control samples. Indeed, these two dominant taxa accounted for more than 90% of fungi in all samples. This finding is consistent with the fungal diversity present in the human environment and may reflect the fact that gastric tissue mycobiota constituents derive from continuous ingestion and inhalation of saprophytic yeasts and molds 9, 10.

The authors further suggested that a gastric fungal imbalance was associated with GC, since principal component analysis indicated that the GC and control groups aggregated separately. Deeper taxonomic coverage of fungal communities revealed a marked increase in the genera *Candida* and *Alternaria* in GC tissue samples compared to non-cancerous control samples, while other genera such as *Saitozyma* (formerly *Cryptococcus*) and *Thermomyces* were found to be less abundant in cancerous tissue. Interestingly, further statistical analysis that was aimed at identifying GC fungal indicators suggested that the observed mycobiota dysbiosis was mainly due to a single yeast species, *Candida albicans*, usually a commensal species in the human gut 11, 12 (Figure 1). It is possible that the substantial increase of this normal microbiota constituent occurred to the detriment of the global gastric mycobiota diversity observed in control samples. Further experiments, such as culture-based methods using GC samples, are needed to verify the increase of *C. albicans* burden. Indeed, many fungal genera that are commonly found in the human environment and thus daily inhaled or ingested were less abundant in GC samples (i.e. *Aspergillus* and *Penicillium* for instance). This raises the important question of whether *C. albicans* mediates GC by reducing the diversity and richness of fungi in the stomach or directly contributes to the pathogenesis of GC. Finally, Zhong and colleagues provide a solid statistical analysis indicating that *C. albicans* had an obvious effect in distinguishing GC and control samples and, as a consequence, could be harnessed as a potential biomarker of diseased tissue.

In conclusion, the research groups of Yongxi Song and Xuehui Hong provide evidence that characterization of the mycobiota ecosystem in stomach tissue may help in distinguishing GC from non-cancerous lesions, though it remains an open question whether *C. albicans* and other reported indicator species are found as a consequence of gastric carcinogenesis or contribute as one of many factors to GC development. Recent advances suggest that in particular pathophysiological contexts, microbial populations associated with the gastrointestinal tissue or mucosa are not well reflected in paired fecal specimens. As a consequence, direct profiling of tissue microbiota is often the sole relevant strategy to shed light on specific dysbiosis states associated with health or diseased tissue microenvironments 13. In this respect, it would be interesting to compare high-throughput sequencing data from both paired biopsies and fecal samples from a new cohort of GC patients. Beyond these technical considerations, this study adds to recent reports that reveal an association of mycobiota dysbiosis and tumorigenesis of the gastrointestinal tract. Aykout and colleagues found that the basidiomycete *Malassezia,* typically a component of the human skin flora 14, is a dominant constituent in the mycobiota during the development of pancreatic ductal adenocarcinoma 5. In the same vein, Zhu and colleagues provided recent insight into an association between mycobiota dysbiosis, immunomodulation, and tumorigenesis in mouse gut 7. More specifically, the investigators reported that *C. albicans* triggered inflammatory IL‐7 secretion by subepithelial macrophages and a metabolic shift toward glycolysis. In turn, these events induced IL‐22 secretion by innate lymphoid cells and promoted colorectal cancer development. Taken together, these recent advancements provide emerging evidence that certain commensal fungi (e.g., *Candida*, *Malassezia*) may modulate local inflammation that is associated with carcinogenesis at different sites of the gastrointestinal tract (Figure 1). This new paradigm not only highlights the potential importance of fungi in the pathogenesis of certain cancers, but also may inform a theoretical basis for the development of diagnostic, prevention, and treatment strategies.

## Figures and Tables

**Figure 1:**
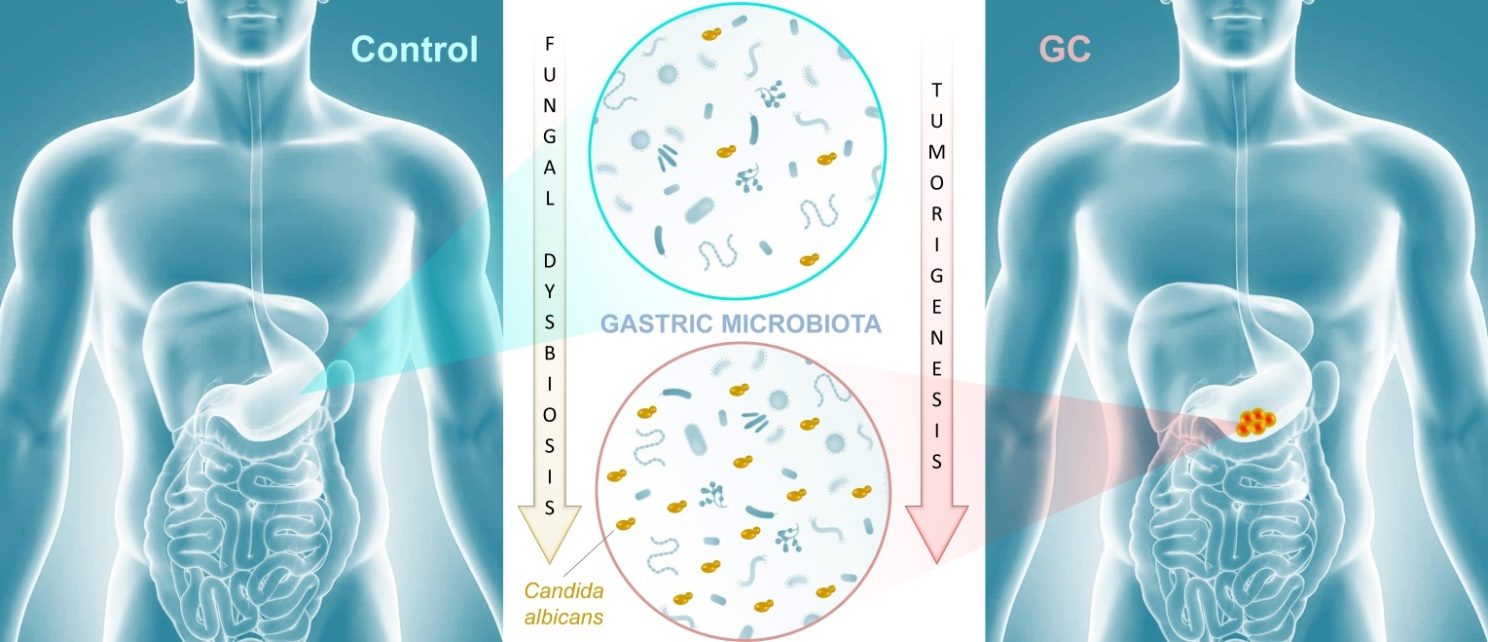
** Mycobiota dysbiosis: a new nexus in gastric tumorigenesis.** Research groups of Yongxi Song and Xuehui Hong took advantage of surgical biopsies that included cancerous lesions and adjacent non-cancerous tissues from 45 patients admitted at the First Affiliated Hospital of China Medical University, Shenyang, China. They provide evidence that characterization of the mycobiota ecosystem in stomach tissue may help in distinguishing gastric cancer from non-cancerous lesions. In particular, statistical analysis aiming at identifying GC fungal indicators suggested that the observed mycobiota dysbiosis in diseased tissue is mainly due to a single yeast species, *Candida albicans*.
